# Agar-Based Resistive Switching Memory for Neuromorphic Applications

**DOI:** 10.1021/acsomega.5c10463

**Published:** 2026-02-12

**Authors:** Han-Chiang Chen, I-Chieh Kao, Keng-Jui Lai, Chun-Yen Chen, Yu-Chi Chang

**Affiliations:** † Department of Engineering Science, 34912 National Cheng Kung University, Tainan City 701, Taiwan; ‡ Research Center for Circular Economy, 34912 National Cheng Kung University, Tainan 701, Taiwan

## Abstract

With the growing demand for sustainable and brain-inspired electronics, biobased resistive random-access memory (Bio-RRAM) has emerged as a promising alternative to conventional inorganic devices. However, most reported Bio-RRAMs still suffer from formation requirements, unstable ON/OFF ratios, or complex hybrid structures, limiting their practical use in neuromorphic computing. Here, we demonstrate a potassium-doped agar Bio-RRAM that overcomes these challenges through a simple, fully biodegradable design. The optimized device achieves forming-free bipolar switching, a stable ON/OFF ratio over 10^3^, stable and reproducible switching cycles, and retention beyond 10^4^ s. More importantly, the device also exhibits synaptic plasticity behaviors, including potentiation, depression, paired-pulse facilitation and depression, and multilevel conductance states, highlighting its feasibility for neuromorphic computing. These findings demonstrate that agar-based bioelectronics can serve as a promising route toward next-generation sustainable memory systems.

## Graphical abstract

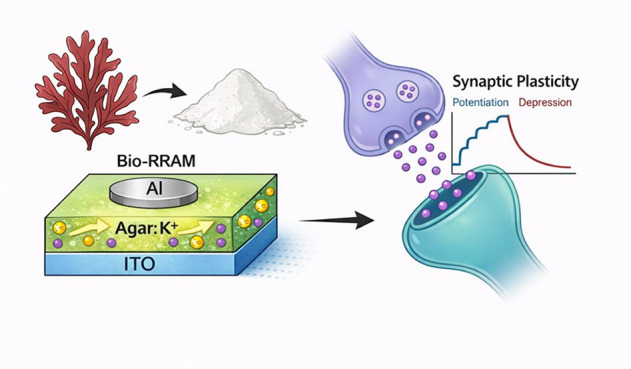

## 1 Introduction

The accelerated upgrade cycles of modern electronics have resulted in the generation of over 50 million tons of e-waste annually, much of which contains nonbiodegradable polymers and hazardous metals. Beyond environmental hazards and resource depletion, this trend underscores the urgent need for sustainable and biodegradable materials in next-generation electronics. Such materials not only mitigate long-term waste accumulation and reduce reliance on rare metals but also provide intrinsic biocompatibility, opening opportunities for implantable biomedical devices, where conventional inorganic materials face integration challenges.

Among emerging nonvolatile memories, resistive random-access memory (RRAM) has emerged as a leading candidate due to its simple metal–insulator–metal structure, high scalability, low operating voltage, and ability to emulate synaptic plasticity for neuromorphic computing. Critically, the explosive growth of big data, artificial intelligence, and brain-inspired computing demands memory technologies with faster speed, lower power, and higher density than conventional flash memory or dynamic random-access memory (DRAM) can provide. RRAM not only fulfills these requirements but also offers complementary metal-oxide-semiconductor (CMOS) process compatibility, enabling seamless integration with existing semiconductor platforms and significantly reducing large-scale production costs. These combined advantages make the development of RRAM essential for future sustainable, high-performance, and intelligent electronics.

With the rise of green electronics and sustainable development concepts, biobased materials have been actively introduced into the field of RRAM, giving birth to a new research direction known as biobased RRAM (Bio-RRAM). These attempts have demonstrated that Bio-RRAM not only realizes fundamental resistive switching characteristics but also offers advantages such as environmental friendliness, low-cost processing, and biocompatibility. For instance, memory devices fabricated from dead leaves, chitosan, silk fibroin, pectin, and egg albumen have successfully exhibited nonvolatile switching behavior and good resistive memory properties, confirming the feasibility and development potential of Bio-RRAM.^1–5^

Building on these promising results, some studies have sought to extend Bio-RRAM toward artificial synaptic emulation, reporting multilevel resistance states and synaptic-like behaviors. However, certain Bio-RRAM achieve high ON/OFF ratios (∼10^4^–10^5^) but operate only in the write-once-read-many (WORM) mode, preventing the rewritable functionality required for synaptic learning.^6,7^ Others demonstrate synaptic plasticity but are constrained by a narrow ON/OFF ratio and limited stability, which hinders stable weight updates.^8^ Therefore, while previous efforts have confirmed that Bio-RRAM is feasible and advantageous in terms of sustainability and eco-friendliness, its broader application in neuromorphic computing and biomedical electronics is still hampered by the lack of rewritable operation and reproducible memory performance and fails to deliver reliable writability with robust synaptic functionality.

These gaps underscore the urgent need for new material strategies that can simultaneously ensure sustainability, process simplicity, high switching stability, and neuromorphic compatibility, thereby advancing Bio-RRAM from proof-of-concept demonstrations toward practical applications in wearables, Internet of Things (IoT) devices, and eco-friendly intelligent electronics.

To address these limitations, this study introduces a biobased RRAM device employing agar, a natural polysaccharide, as the switching medium, further enhanced through potassium ion (K^+^) doping to improve conductive filament control and resistive switching stability. Agar provides eco-friendliness, biodegradability, and biocompatibility, while K^+^ ions contribute ionic pathways for more reliable switching dynamics. As a result, the fabricated agar:K devices achieve forming-free bipolar switching, a stable ON/OFF ratio of ∼10^3^, and retention exceeding 10^4^ s under ambient conditions. Moreover, they exhibit synaptic functionalities such as multilevel resistance states, long-term potentiation and depression, and paired-pulse facilitation and depression, highlighting their potential for both sustainable electronics and neuromorphic applications. These unique advantages underscore the promise of agar-based resistive switching devices as a feasible pathway toward eco-friendly, CMOS-compatible, and low-cost nonvolatile memory solutions, paving the way for future biocompatible electronics and brain-inspired computing architectures.

## 2 Experiments

### 2.1 Preparation of Agar and K^+^-Doped Agar Solutions

Indium tin oxide (ITO)-coated glass substrates (1.5 × 2.0 cm^2^) were ultrasonically cleaned in acetone, methanol, and deionized water for 10 min each to remove surface organic contaminants and particulate residues. The agar solution was prepared by dissolving 0.1 g of agar powder in 5 mL of deionized water, corresponding to a concentration of 2 wt % agar. For the potassium-doped agar formulation (AGK), 0.025 g of KNO_3_ was added to 5 mL of the same agar solution, yielding a KNO_3_ concentration of approximately 0.5 wt %. The mixtures were stirred on a magnetic hot plate at 150 °C for 30 min to ensure complete dissolution and homogeneous mixing.

### 2.2 Thin-Film Deposition and Device Fabrication

The prepared agar solutions were spin-coated onto precleaned ITO/glass substrates at 3000 rpm for 60 s with an acceleration rate of 500 rpm/s. The coated substrates were subsequently baked on a hot plate at 80 °C for 10 min to promote solvent evaporation and initial film solidification. After film formation, aluminum (Al) top electrodes with a thickness of approximately 200 nm were deposited through a metal shadow mask via thermal evaporation, yielding Al/agar (or K^+^-agar)/ITO device structures for electrical characterization. The devices consisted of circular Al top electrodes with a diameter of 200 μm, corresponding to an active device area of 3.14 × 10^–4^ cm^2^. The thickness of the agar or agar:KNO_3_ switching layer was approximately 136 nm, as determined from focused ion beam (FIB) cross-sectional analysis.

### 2.3 Material Characterization

Atomic force microscopy (AFM, Dimension ICON with NanoScope V controller, Bruker, USA), operated in tapping mode with a scan size of 5 × 5 μm^2^ was used to evaluate surface morphology and root-mean-square (RMS) roughness. Surface analysis was performed using NanoScope software (Veeco, USA). X-ray photoelectron spectroscopy (XPS) measurements were carried out by using a monochromatic Al Kα source (1486.6 eV). High-resolution spectra were collected with a pass energy of 20 eV, and all binding energies were calibrated to the C 1s peak at 285.0 eV. Crystalline structure and phase composition were examined by using X-ray diffraction (XRD). Cross-sectional microstructures were analyzed by using FIB (NOVA 600 NanoLab, FEI) milling. Elemental distribution was further characterized by scanning electron microscopy equipped with energy-dispersive X-ray spectroscopy (SEM-EDS).

### 2.4 Optical and Electrical Measurements

Optical transmittance of the agar-based thin films was evaluated by using UV–visible spectrophotometry. All electrical measurements were performed under ambient conditions by using a Keithley 4200-SCS semiconductor parameter analyzer connected to a probe station. During *I–V* characterization, the ITO bottom electrode was grounded, while the voltage sweep was applied to the top Al electrode. Direct-current *I*–*V* sweeps were conducted to evaluate the resistive switching characteristics and synaptic emulation behavior of the fabricated Bio-RRAM devices.

## 3 Results and Discussion

To verify the incorporation of potassium ions into the agar films, XPS survey spectra of pure agar (AG) and agar mixed with 0.025 g of KNO_3_ (AGK) films were collected, as shown in Figure 1a. In the AG film (black curve), the dominant peaks correspond to C 1s (∼285 eV) and O 1s (∼532 eV), consistent with the polysaccharide backbone structure of agar. A weak N 1s peak (∼400 eV) was also observed, originating from residual amino-containing groups in the natural agar. Importantly, no significant potassium signal was detected in AG, with the measured K 2p content being <0.1 at. %. In contrast, the AGK film (red curve) exhibited two additional peaks at ∼293 eV and ∼296 eV, which can be assigned to K 2p_3/2_ and K 2p_1/2_, confirming the presence of K^+^ ions in the +1 oxidation state.^9^ The atomic composition analysis further revealed a potassium content of 9.4 at. %, accompanied by a relative decrease in carbon content (from 62.4 to 49.3 at. %) and an increase in oxygen content (from 37.5 to 41.2 at. %). This increase in oxygen intensity can be attributed to the contribution of nitrate (NO_3_^–^) groups from KNO_3_, further supporting the successful chemical incorporation of potassium nitrate into the agar matrix. Figure 1b–d presents the high-resolution spectra of the K 2p_3_, C 1s, N 1s, and O 1s regions, respectively. The high-resolution spectra clearly confirm the incorporation of potassium ions through the characteristic K 2p_3/2_ and 2p_1/2_ peaks, while the nitrate-related N 1s signal at approximately 406.5 eV verifies the presence of NO_3_^–^ originating from KNO_3_. The O 1s components further distinguish lattice-related oxygen from chemisorbed oxygen species, and the C 1s peaks reveal the functional groups within the agar backbone. These chemical states provide direct evidence of potassium incorporation and defect modulation within the agar matrix, thereby supporting the proposed ionic conduction mechanism.^10^ Therefore, the survey spectra and atomic composition analysis confirm that KNO_3_ doping effectively introduces potassium ions into the agar films while preserving the fundamental C–O–N framework. The incorporation of K^+^ not only modifies the chemical environment but also provides ionic pathways that are expected to regulate the formation of conductive filaments and enhance the switching uniformity of Bio-RRAM devices.

**Figure 1:**
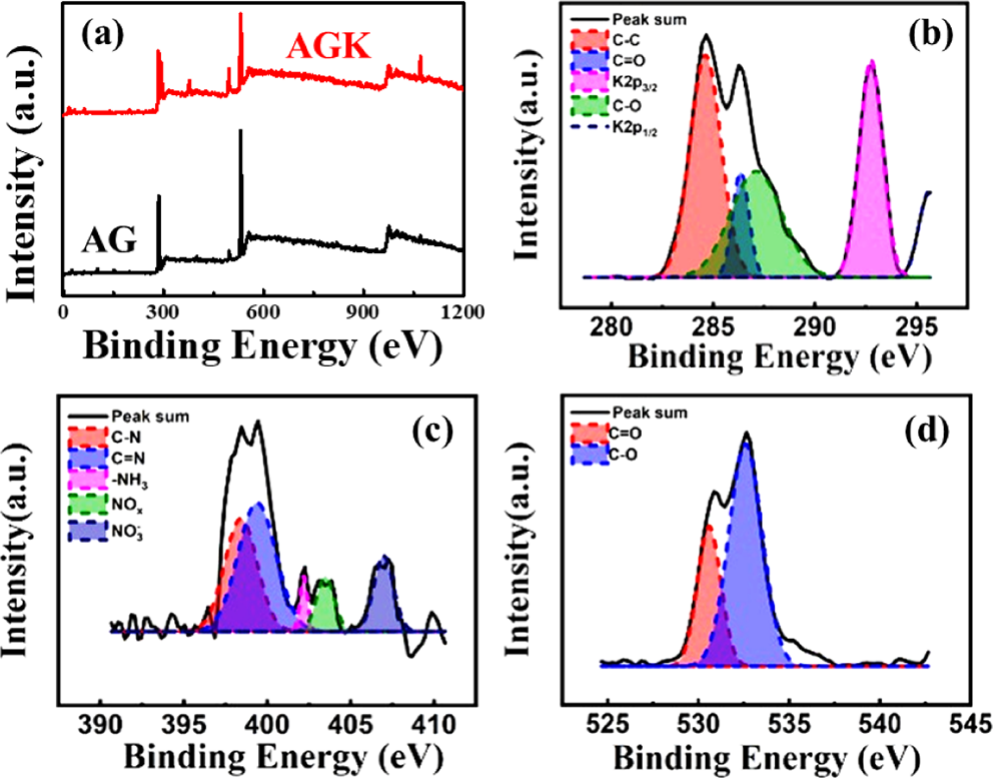
(a) High-resolution XPS spectra of AG and AGK thin films. High-resolution XPS spectra and peak fitting results of (b) K 2p, C 1s, (c) N 1s, and (d) O 1s for the AGK thin film.

To investigate the effects of potassium ions on the surface morphology and roughness of the agar films and realize stable and reproducible resistive switching in Bio-RRAM, the dielectric film must maintain a smooth and uniform surface since excessive roughness or structural instability may cause stochastic filament formation and poor device reliability. Figure 2a and b presents two-dimensional atomic force microscopy (AFM) images of AG and AGK, respectively. The AG film exhibited a uniform and fine surface morphology with an *R*_rms_ of 2.97 nm, while the AGK film showed a reduced *R*_rms_ of 2.41 nm, suggesting that potassium ions help stabilize the matrix and suppress surface irregularities. These results indicate that controlled KNO_3_ doping can help stabilize the film structure and further reduce surface roughness.

**Figure 2:**
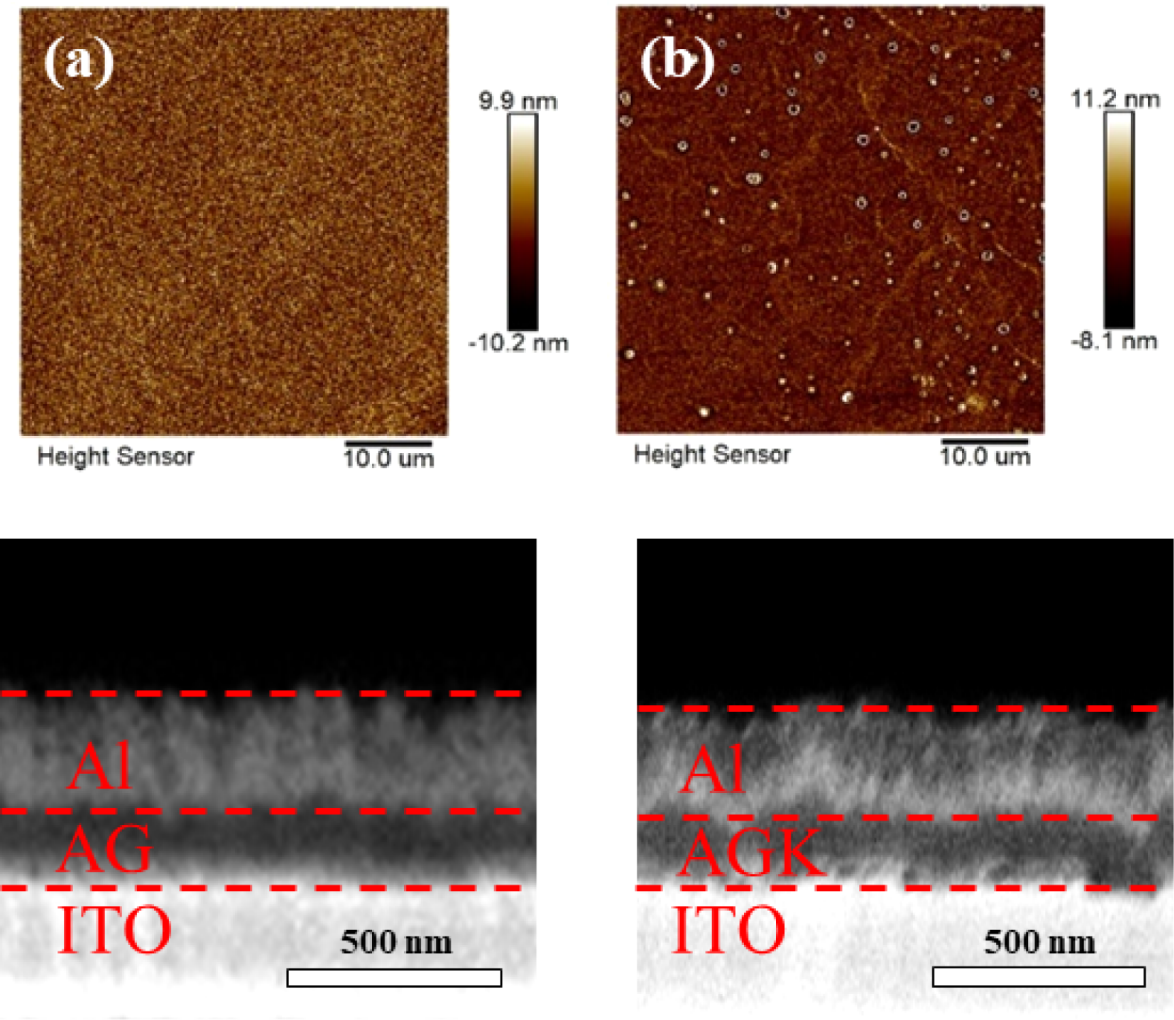
2D AFM images of the (a) AG and (b) AGK thin films. FIB cross-sectional images of (c) AG and (d) AGK memory devices.

To ensure that the doping strategy does not compromise the physical thickness or continuity of the films, we examined focused ion beam (FIB) cross-sections. Figure 2c and d shows that both AG and AGK memory devices exhibited comparable thicknesses (∼136 nm), confirming that small doping amounts do not alter film deposition or induce structural collapse. This result is critical, as it shows that ionic modification can be achieved without introducing variability into device geometry, which is essential for scalable memory fabrication.

Having confirmed that potassium doping improves film smoothness and chemical stability, we also examined its impact on resistive switching performance. Figure 3a and b shows the *I–V* characteristics of the devices based on AG and AGK, respectively. During the electrical measurements, a bidirectional voltage sweep of 5 V → 0 V → −5 V → 0 V → 5 V was applied to the devices. The AGK device exhibited a forming-free bipolar resistive switching behavior. The SET voltage (*V*_SET_) was approximately −2.2 V, the RESET voltage (*V*_RESET_) was approximately 3 V, and the ON/OFF ratio was over 10^3^. Compared to the AG memory device, which displayed no resistive switching behavior, the doped system demonstrated improved filament regulation due to ionic contributions from K^+^.

**Figure 3:**
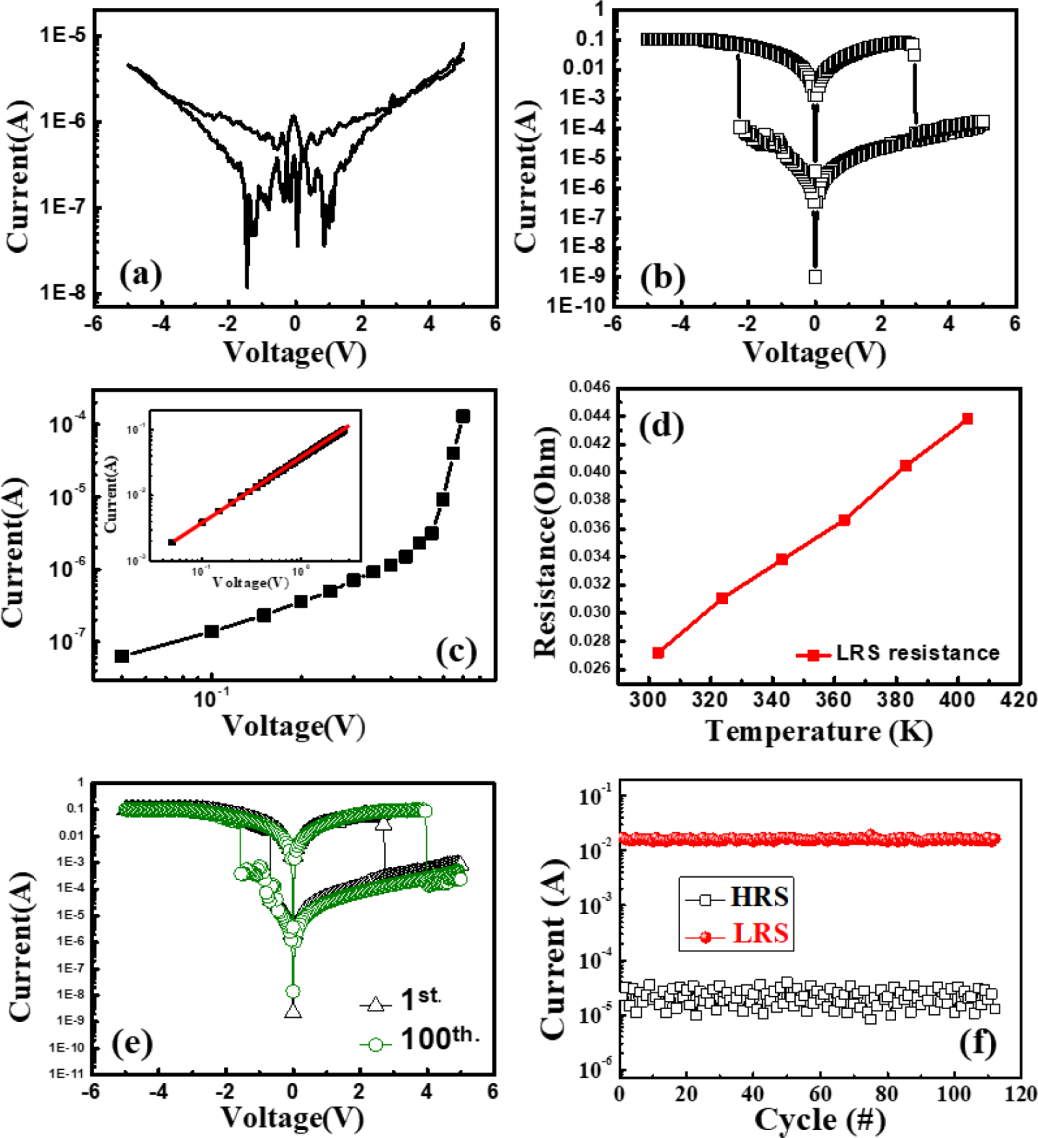
*I–V* curve of (a) AG and (b) AGK memory devices. (c) Linear fitting results of the *I–V* curve for the AGK memory device in the HRS. The inset shows the linear fitting behavior of the AGK memory device in the LRS. (d) Resistance variation of the LRS at different temperatures, (e) switching cycle characteristics, and (f) endurance characteristics for the AGK memory device.

To further investigate the conduction mechanism, a log–log fitting analysis of the *I–V* curve was performed for the AGK memory device, as shown in Figure 3c. The log–log *I*–*V* plots indicate three distinct conduction regions. At low voltages, the current exhibits an ohmic dependence (slope ≈ 1), while at intermediate voltages, the slope increases (>1), suggesting trap-controlled transport. At higher voltages, the slope further increases, consistent with a trap-regulated space-charge-limited conduction (SCLC) process. These observations are in line with typical SCLC behavior reported in the literature.^11–14^

The agar:KNO_3_ device exhibits formation-free bipolar switching. Under negative bias (SET), K^+^ ions and defect states promote the formation of conductive filaments, while positive bias (RESET) leads to their partial rupture. Log–log *I–V* analysis shows ohmic behavior at low voltages and trap-controlled SCLC at higher voltages, indicating trap-assisted filament conduction. Overall, the switching arises from the reversible modulation of ion- and defect-assisted conductive filaments within the agar matrix.

The incorporation of K^+^ ions may influence the trap landscape within the agar matrix; however, the exact microscopic mechanism requires further investigation. Here, the improved switching uniformity is attributed phenomenologically to the presence of ionic species, without assigning a specific conduction pathway.

To confirm the composition of the conductive filaments within the device, temperature-dependent resistance measurements of the low-resistance state (LRS) were conducted for the AGK memory device. The measurement temperature range was from 30 °C to 120 °C, with a read voltage set at 0.1  V. The variation of resistance with temperature was plotted. As shown in Figure 3d, the LRS resistance increased with rising temperature, which is characteristic of typical metallic conduction behavior.^15,16^ Furthermore, the temperature coefficient of resistance (TCR) was calculated using the following equation: *R*(*T*) = *R*_0_ [1 + α (*T* – *T*_0_)], where *R*_0_ is the resistance at the reference temperature *T*_0_, and α is the temperature coefficient of resistance. Based on data fitting analysis, the TCR value for the AGK memory device was determined to be approximately 6.1 × 10^–3^ K^– 1^, with a positive value, further confirming that the conductive paths are primarily composed of metallic conductive filaments.^17–20^

Figure 3e shows the switching cycle performance of the AGK memory device. After 100 consecutive switching cycles, the *I–V* characteristics and switching behavior of the device remained stable, with minimal variation in the ON/OFF ratio, demonstrating excellent cycling endurance and operational repeatability. These results indicate that the AGK memory device exhibits high stability and outstanding durability, making it suitable for long-term and frequent memory operation applications.

Figure 3f shows the endurance characteristics of the AGK device. Endurance was measured under DC voltage sweeps. The AGK device sustained its stability for up to 112 cycles. The current value during the endurance test was read at a voltage of 0.5 V. Despite the limited number of cycles, the AGK device showed stable switching behavior.

To evaluate stability, statistical analysis of currents and voltages was performed. Figure 4a shows the cumulative probability distribution of currents in the high resistance state (HRS) *I*_HRS_ and *I*_LRS_ for the same device. The coefficients of variation (CV) were 17% for *I*_HRS_ and 11% for *I*_LRS_, respectively. The inset of Figure 4a presents the statistical distribution of *V*_SET_ and *V*_RESET_ for the AGK memory device, which is aimed at evaluating the switching voltage stability. Statistical analysis was conducted across 25 devices. Similarly, both voltages showed concentrated distributions, reflecting good repeatability and operational stability. This stability may be attributed to the beneficial ionic contribution of K^+^ ions and the defect engineering effect from agar templating.

**Figure 4:**
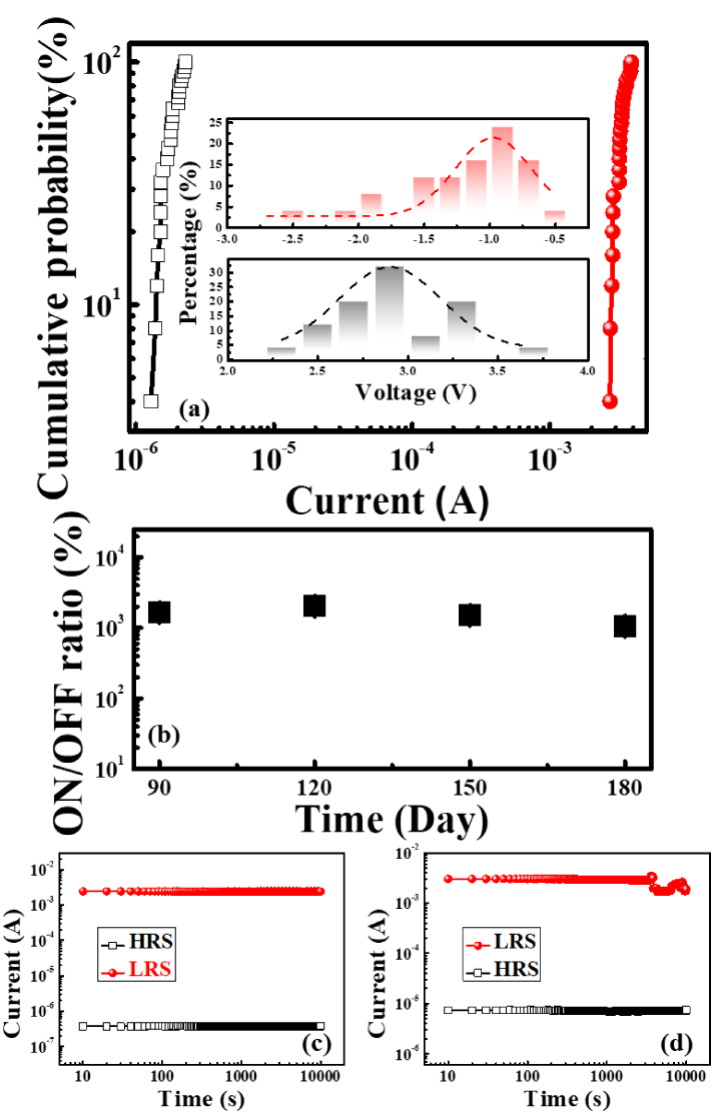
(a) Statistical results for the current and voltage of the AGK memory device. (b) Variation of the ON/OFF ratio over time for the AGK memory device. Retention characteristics of the AGK resistive switching device were measured (c) at room temperature and (d) at 85 °C.

Long-term stability tests were conducted on the AGK memory device under ambient conditions at room temperature, with the ON/OFF ratio measured and recorded every 30 days, as shown in Figure 4b. The results indicate that, although the ON/OFF ratio exhibited a gradual decline over time, it remained above 10^3^ even after 180 days. This result demonstrates that the AGK memory device maintains excellent environmental stability and long-term operational reliability, even after prolonged exposure to air.

To further evaluate the data retention performance, long-term stability tests were conducted at room temperature, and the results are shown in Figure 4c. The current levels of the HRS and LRS were monitored under a read voltage of 0.1 V. Experimental results confirmed that both the HRS and LRS currents remained stable over a period exceeding 10^4^ s, with no significant degradation or failure observed. As illustrated in Figure 4d, the accelerated retention test was conducted at 85 °C. The distinction between HRS and LRS remained stable throughout the test duration, supporting the robustness of the device under thermal stress. These findings verify the excellent long-term data retention capability and reliability of the AGK memory device, demonstrating its potential for long-term operation in nonvolatile memory applications.

To verify the potential of the AGK memory device for high-density data storage and neuromorphic computing applications, current measurements were performed on the HRS and LRS of the device. The tests were conducted under a read voltage of 0.1 V, and current variations during multiple switching cycles were observed. To explore the multilevel storage capability of the device, different current compliance (CC) conditions—0.1 0.01, and 0.001 A—were applied, and their effects on the LRS resistance states were compared. Figure 5a shows that with varying CC levels, the AGK memory device exhibited clearly distinguishable and controllable gradations in the LRS current values, with stable separation between each resistance level and without significant overlap or fluctuation. This demonstrates the excellent multilevel memory characteristics of the device. The ability to stably modulate multiple resistance states can effectively increase storage density beyond the limitations of traditional binary storage designs, offering a viable solution for future high-performance memory devices. Moreover, the continuous tunability of multilevel conductance is analogous to the adjustment of synaptic weights in biological synapses, enabling the emulation of long-term potentiation (LTP) and long-term depression (LTD) behaviors.

**Figure 5:**
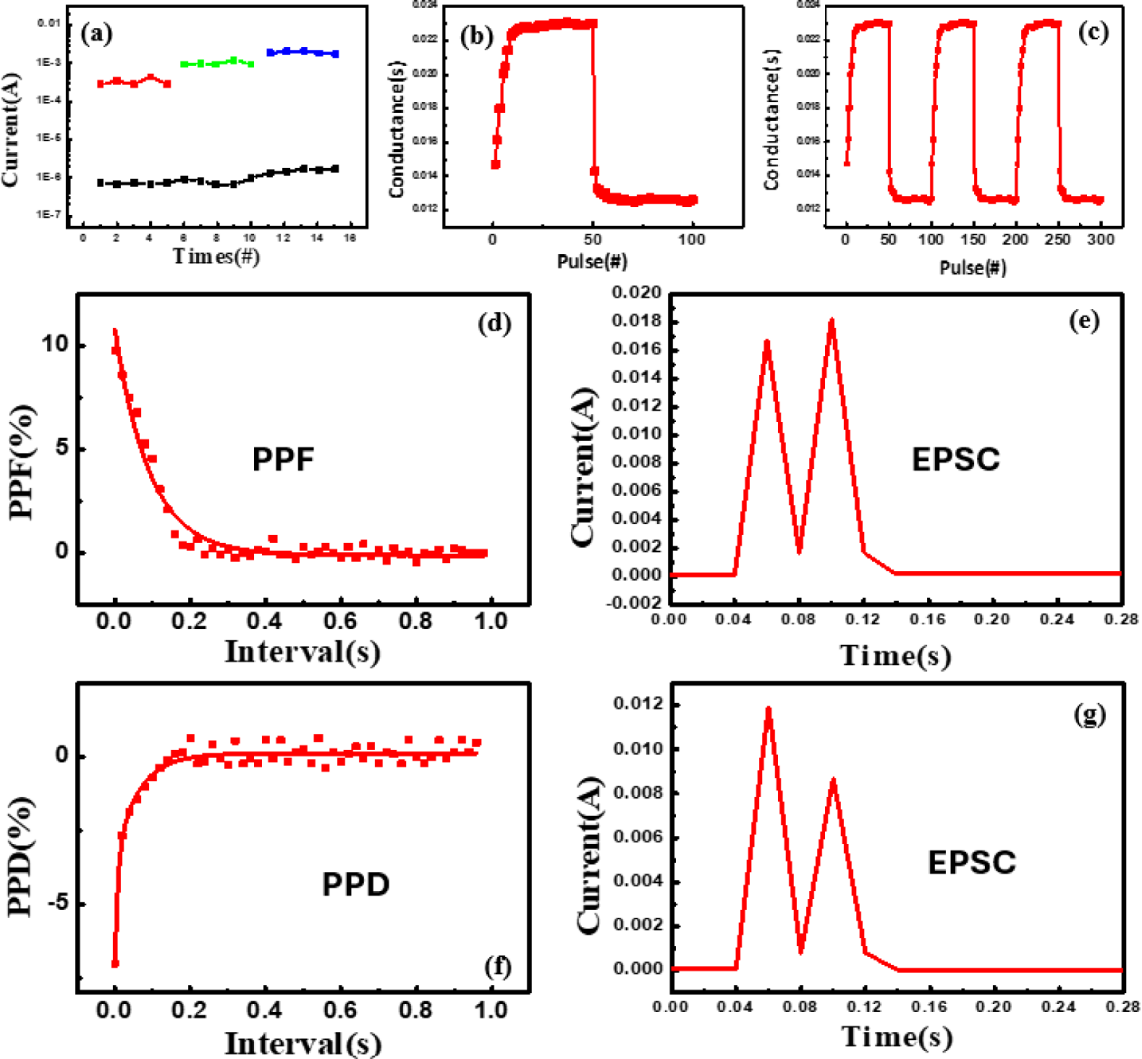
(a) Multilevel storage test of the AGK memory device under different CC conditions. Conductance variation curves (b) during synaptic potentiation and depression and (c) after three consecutive potentiation and depression pulse cycles of the AGK memory device. (d) Paired-pulse facilitation (PPF) response and fitting curve, (e) excitatory postsynaptic current (EPSC) curve, (f) paired-pulse depression (PPD) response and fitting curve, and (g) excitatory postsynaptic current (EPSC) curve of the AGK memory device.

Figure 5b further illustrates the potentiation and depression behaviors of the AGK memory device, simulating the plasticity characteristics of biological synapses. Initially, 50 potentiation pulses were applied, resulting in a gradual increase in device conductance, displaying a linear upward trend. Subsequently, 50 depression pulses were applied, leading to a gradual decrease in conductance, mimicking the signal suppression process observed in biological synapses. During the test, the applied voltages for the potentiation and depression pulses were set to −1.1 and 1.1 V, respectively, while the read pulse voltages were set to −0.1 and 0.1 V, respectively, ensuring that the conductance state was not disturbed during the read process. Each pulse had a width of 1 ms with an interval time of 10 ms, enabling stable and precise observation of the conductance variations. To verify the repeatability of the AGK memory device, three potentiation and depression pulse cycles were applied. Each cycle consisted of 50 potentiation pulses and 50 depression pulses, simulating the long-term potentiation and LTD behaviors of synapses to evaluate the stability and conductance variation characteristics of the device under continuous pulse stimulation. As shown in Figure 5c, after three complete cycles of potentiation and depression, the conductance variation curves of the device remained highly consistent, with no significant degradation or instability observed. This demonstrates that the AGK memory device effectively emulates synaptic potentiation and depression, demonstrating excellent reversibility and repeatability. These results confirm its stable potential for neuromorphic computing and the future development of memory devices with synaptic plasticity.

To investigate the short-term synaptic characteristics of the AGK device, paired-pulse facilitation (PPF), excitatory postsynaptic current (EPSC), paired-pulse depression (PPD), and inhibitory postsynaptic current (IPSC) measurements were conducted. PPF and EPSC correspond to excitatory responses, where the second pulse induces a larger current due to residual ionic motion. In contrast, PPD and IPSC reflect inhibitory behavior, where the second pulse produces a reduced current. Together, these measurements indicate that the device can emulate biologically relevant short-term plasticity under both excitatory and inhibitory stimuli.

As shown in Figure 5d, PPF measurement was performed, and the facilitation ratio was calculated using the formula PPF = (*I*2 – *I*1)/*I*1 × 100%.^21,22^ When two identical pulses (−1.1 V, 2 ms) were applied, the current triggered by the second pulse (*I*2) was larger than that triggered by the first pulse (*I*1), demonstrating a clear facilitation effect. As the time interval (Δ*t*) between the two pulses increased, the PPF value gradually decreased, consistent with the characteristic behavior of short-term enhancement in biological synapses. Figure 5e presents the performance of the AGK memory device under EPSC testing. After two consecutive pulses (−1.1 V, 2 ms), the current induced by the second pulse was significantly higher than that induced by the first pulse, indicating that the device exhibits excitatory characteristics and successfully mimics the natural signal enhancement response process of biological synapses.

As shown in Figure 5f, when two identical depression pulses (1.1 V, 2 ms) were applied to the AGK memory device, the current response triggered by the second pulse was significantly reduced. This phenomenon was quantified using the PPD formula: PPD = (*I*2 – *I*1)/*I*1 × 100%.^19,20^ As the time interval (Δ*t*) between the two pulses increased, the PPD value exhibited a continuous decline, reflecting the typical characteristics of short-term depression (STD) observed in biological synapses and consistent with short-term plasticity (STP) behavior. Figure 5g presents the EPSC characteristics of the AGK memory device. When two consecutive excitatory pulses (1.1 V, 5 ms) were applied, the current response induced by the second pulse was slightly lower than that of the first pulse, reflecting typical excitatory postsynaptic behavior.

Although the device exhibits stable conductance modulation under repeated potentiation and depression pulses, these results represent a short-term conductance evolution rather than long-term potentiation. Additional retention tests of intermediate conductance states are required to confirm long-term potentiation behavior, as demonstrated in previous literature.^23,24^

Although some Bio-RRAMs report ON/OFF ratios of over 10^3^,^25,26^ the AGK device achieves stable, rewritable, forming-free bipolar switching with reliable cycle-to-cycle statistics and synaptic plasticity within a fully biodegradable polysaccharide matrix, which remains comparatively rare among biodegradable materials.^1,2,4,26^

Table 1 summarizes and compares the resistive switching performance of the Al/AGK/ITO device with previously reported biobased RRAM devices.

**Table 1:** Comparison of Al/AGK/ITO with the Reported Data for Memory Devices

Structure	ON/OFF ratio	Retention	*V*_set_/*V*_reset_	Ref
Al/DR1-PDPAF-DR1/ITO	10^3^	3600	3.5/–1	^25^
Ag/Chitosan + Ag/Pt	10^5^	10^4^	0.4/–0.48	^26^
Ag/leaves/Ti	10	10^3^	1/–1.5	^1^
Ag/Pectin/FTO	10^2^	10^3^	3.3/–4.5	^4^
Mg/Chitosan + Ag/Mg	10^2^	10^4^	1.6/–0.8	^2^
Al/Silk/ITO	10	10^3^	10.4/–11.5	^2^
Al/AGK/ITO	10^3^	10^4^	–2.2/3	This work

This response demonstrates that the AGK memory device can effectively emulate biologically relevant excitatory synaptic dynamics and short-term plasticity. These results further indicate that the device reproduces key short-term synaptic functions, laying a solid foundation for its potential applications in neuromorphic computing and next-generation synaptic biomimetic devices.

## 4 Conclusion

This study demonstrated that potassium ion doping is an effective strategy to stabilize agar films and enhance their resistive switching behavior. At an optimal KNO_3_ concentration of 0.025 g, the films retained smooth morphology and chemical stability while enabling forming-free bipolar switching, ON/OFF ratios exceeding 10^3^, stable retention over 10^4^ s, and endurance across 100 cycles. The device further emulated synaptic behaviors, including PPF, PPD, LTP, and LTD, underscoring its potential for neuromorphic computing. Overall, agar combined with appropriate potassium ion doping provides a sustainable and high-performance platform for flexible memory devices, offering a promising route toward eco-friendly electronics and brain-inspired architectures.

## Abbreviations

RRAM: resistive random-access memory
Bio-RRAM: bio-based resistive random-access memory
AG: agar film (pure)
AGK: potassium-doped agar film
KNO_3_: potassium nitrate
ITO: indium tin oxide
AFM: atomic force microscopy
XPS: X-ray photoelectron spectroscopy
FIB: focused ion beam
HRS: high resistance state
LRS: low resistance state
SCLC: space-charge-limited current
TFLC: trap-filled-limited current
CC: current compliance
LTP: long-term potentiation
LTD: long-term depression
STP: short-term plasticity
PPF: paired-pulse facilitation
PPD: paired-pulse depression
EPSC: excitatory postsynaptic current
IPSC: inhibitory postsynaptic current
TCR: temperature coefficient of resistance
